# Impact of ultrasonic dispersion on the photocatalytic activity of titania aggregates

**DOI:** 10.3762/bjnano.6.250

**Published:** 2015-12-17

**Authors:** Hoai Nga Le, Frank Babick, Klaus Kühn, Minh Tan Nguyen, Michael Stintz, Gianaurelio Cuniberti

**Affiliations:** 1 Institute for Materials Science and Max Bergmann Center of Biomaterials, TU Dresden, 01062 Dresden, Germany; 2 School of Chemical Engineering, Hanoi University of Science and Technology, 10000 Hanoi, Vietnam; 3 Institute of Process Engineering and Environmental Technology, TU Dresden, 01069 Dresden, Germany; 4 Dresden Center for Computational Materials Science (DCCMS), TU Dresden, 01062 Dresden, Germany; 5 Center for Advancing Electronics Dresden, TU Dresden, 01062 Dresden, Germany

**Keywords:** AOPs, reaction rate constant, turbidity, ultrasonic energy, wastewater treatment

## Abstract

The effectiveness of photocatalytic materials increases with the specific surface area, thus nanoscale photocatalyst particles are preferred. However, such nanomaterials are frequently found in an aggregated state, which may reduce the photocatalytic activity due to internal obscuration and the extended diffusion path of the molecules to be treated. This paper investigates the effect of aggregate size on the photocatalytic activity of pyrogenic titania (Aeroxide^®^ P25, Evonik), which is widely used in fundamental photocatalysis research. Well-defined and reproducible aggregate sizes were achieved by ultrasonic dispersion. The photocatalytic activity was examined by the color removal of methylene blue (MB) with a laboratory-scale setup based on a plug flow reactor (PFR) and planar UV illumination. The process parameters such as flow regime, optical path length and UV intensity are well-defined and can be varied. Our results firstly show that a complete dispersion of the P25 aggregates is not practical. Secondly, the photocatalytic activity is not further increased beyond a certain degree of dispersion, which probably corresponds to a critical size for which UV irradiation can penetrate the aggregate without significant obscuration.

## Introduction

Advanced oxidation processes (AOPs) form a group of modern chemical technologies that rely on the generation of radical species and are considered to have high prospects for the oxidation, discoloration, mineralization, and degradation of organic pollutants [1–2]. Photocatalysis is an example of an AOP that has been effectively applied for the treatment of highly polluted water such as dye sewage [3–4]. Among the materials for this application, titanium dioxide (TiO_2_) is a very promising photocatalyst because of its commercial availability, chemical and biological inertness, and because it has no known adverse health effects on humans [5–6]. Due to its large active surface area, the suspended TiO_2_ powder is favored [6].

Most slurry photocatalysts have been implemented in illuminated batch reactors [6–8] and follow Langmuir–Hinshelwood kinetics [9–10]. This research has focused on the materials aspects such as the structural properties (e.g., surface area, particle size, crystal composition, porosity) [8,11] of pristine or modified photocatalysts [2,5,12]. However, many of these laboratory-scaled apparatus are inappropriate to be applied in well-defined conditions, making the application with available pilot photoreactors challenging. Besides, such particles often form aggregates [5,13] whose properties differ from those of the primary particles, leading to misconceptions about the photocatalytic characteristics. In contrast to the photochemical aspect, in which aggregation has been more thoroughly discussed [14–15], the effects of the aggregates/secondary particles on the photocatalytic applications are still inexplicit. While the higher photocatalytic activity of fine, primary particles (as a result of the larger surface area) has been investigated [4,16–18], the behavior and properties of the aggregates is not well understood.

This paper shows an engineering approach to study the aggregation in photocatalysts. The first part presents the experimental setup, which defines the process parameters. In addition, ultrasonic dispersion was used to disintegrate the P25 nano-photocatalyst as well as vary the size. The photocatalytic activity was examined by the discoloration of MB under UV irradiation.

## Experimental

### Materials

All experiments were conducted with commercial titanium(IV) oxide powder (Aeroxide^®^ P25, Evonik, CAS-No. 13463-67-7), which consists of an approximately 80/20 w/w rutile/anatase mixture.

MB (Merck, KGaA), a model substance in dye wastewater research [4,7], was chosen as the organic compound in the photocatalysis. The discoloration of MB, in consequence, indicates the photocatalytic properties of P25 [19].

### Experimental setup

An industrial, photocatalytic implementation requires a photoreactor, which not only satisfies basic techniques of chemical engineering [10] but also ensures effective photon collecting [20].

**Figure 1:**
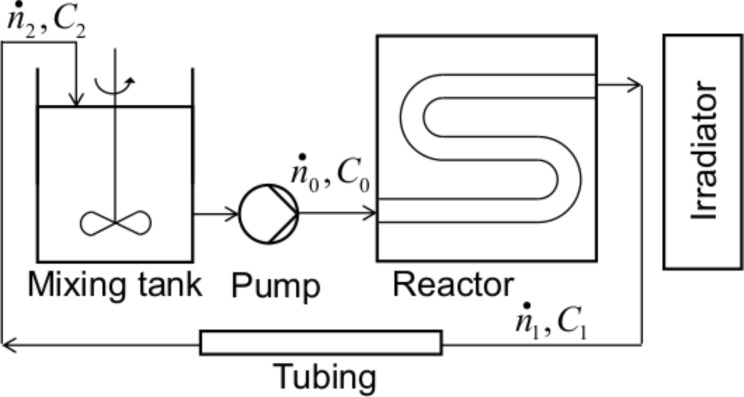
Schematic of the experimental setup consisting of a reservoir, pump, photoreactor and irradiator.

An idea for a simple experimental setup was developed (Figure 1) with the following key points: (1) the artificial illuminator is planar and produces steady-state UV radiation of intensity similar to that of solar illumination (20–30 W/m^2^) [4–5,21]; (2) the reactor is a rectangular cell, so that the influence of the UV intensity is only two-dimensional; (3) the thickness of the reactor is small to diminish the shielding effect in the non-illuminated region [22]; and (4) most importantly, the reactor operates based on a PFR as the absorbent tube in the solar collecting reactors [4,23–26].

**Figure 2:**
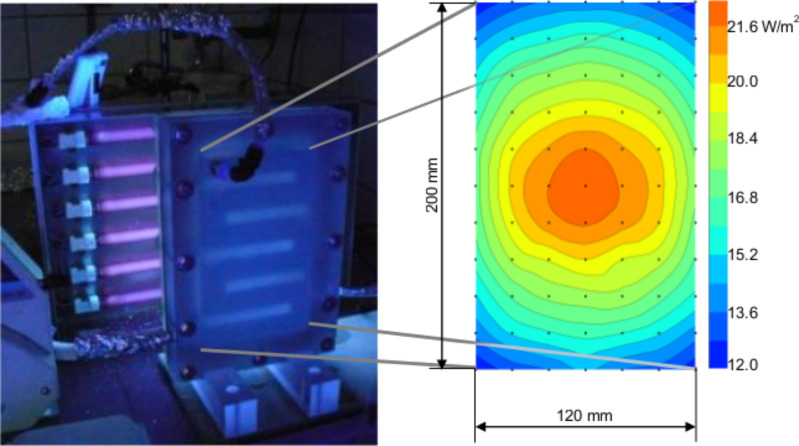
The photoreactor placed in front of the irradiator and the UV intensity distribution on the illuminated surface.

The setup design was then developed as shown in Figure 2. The reactor has a footprint size of 260 × 180 × 35 mm and includes six continuous channels, of which the size is 25 × 120 × 22 mm corresponding to a volume of 411 mL. The illuminated surface of the reactor is 0.45 cm^2^ and is made from 3.3 mm Schott Borofloat^®^ 33 glass, which has a UVA transmittance >90%, as specified by the manufacturer. The illuminating device was constructed by UMEX GmbH with six Phillips 8 W mercury fluorescent tubes with a mode wavelength value of 365 nm. The UV intensity at the window of the reactor measured by an intensity meter (PCE-UV34) was 12.0–22.1 W/m^2^ (Figure 2). The circulation of fluid is driven by a Micropump^®^ 132-665-316 pump allowing a flow rate of 2.82 L/min, corresponding to a Reynolds number in the channels of ≈1150.

The appropriate process parameters such as flow regime, optical path length and average UV intensity are defined and can be adjusted. This setup allows the establishment of other specific constants, such as intensity- or flow-regime-based reaction rate constants for new investigations.

### Experimental determination of reaction rate constant

Langmuir–Hinshelwood kinetics have been commonly applied to quantify the photocatalytic conversion of organic compounds in batch reactors [9–10]. For a new design based on a PFR, determining the reaction rate constant is required.

Since the change of the amount of organic compound A in the PFR is produced only by the reaction, the material balance is derived as [27]:

[1]
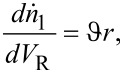


where *n* and 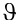 are moles and the stoichiometric coefficient of species A, respectively, *V*_R_ is the volume of the PFR, *r* is the reaction rate equal to *r* = *kC*_1_, and *k* is the reaction rate constant.

In the mixing tank, by assuming that the change of amount is due to the in- and outflows, and not due to the reaction, the material balance follows [27]

[2]
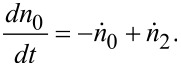


The concentration of species A is specified as

[3]
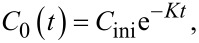


where *K* characterizes the overall degradation of species A in the whole system. The reaction rate constant in the reactor, *k*, is investigated from *K* as

[4]
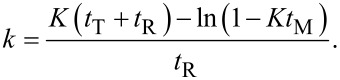


The assumptions as well as more details of the determination of the reaction rate constant can be found in Supporting Information File 1.

### Experimental methods

#### Ultrasonic dispersion

Two ultrasonic processors, Topas UDS751 (sonotrode S7) and Hielscher UP100H (sonotrode MS7), were employed to disperse the 1 g/L TiO_2_ P25 suspensions [28]. The dispersed volumes were varied and the ultrasonic power was altered by varying the amplitude (20–100%) and the immersion level of the sonotrodes (2.0–5.5 cm). The power was measured by a Voltcraf^®^ Energy Logger 4000. Samples were periodically taken for size characterization and transmittance measurement.

#### Color removal of methylene blue

The photocatalytic properties of P25 were examined by characterizing the discoloration of MB. The desired MB concentration [6–7,29] of 0.01 mM was obtained by adding the MB stock solution into the dispersed P25 suspensions. To achieve an adsorption–desorption equilibrium, the suspensions were stirred in dark for 30 min. The discoloration was then performed in the illuminated flow reactor. Samples were taken at one-, five- or ten-minute intervals. The supernatant fluids were separated by an Eppendorf 5417 centrifuge and stored for further analysis.

The process parameters of these experiments were adapted to different purposes: (1) verifying the kinetic model by altering the volume of the suspensions, (2) studying the influence of the P25 photocatalyst size by varying the degree of ultrasonic dispersion, and (3) investigating the effect of the turbidity of the suspensions by working with different P25 concentrations. All experiments were repeated two or three times to check the reproducibility.

#### Analytical method

The particle size distribution of the TiO_2_ P25 suspensions was characterized by a Malvern Nano S90 photon correlation spectrometer [30]. The immediate results are the intensity-weighted distribution functions. Two parameters of analysis, the intensity-weighted harmonic mean size, *x*_cum_, and the polydispersity index, PDI, were examined as a function of dispersion time.

The extinction coefficient of the P25 suspensions was calculated by means of the Beer–Lambert equation from the transmitted light measured with a Varian Cary 100 Bio UV–vis spectrometer [31]. The transmittance was investigated through 10 mm path length P25 suspensions with concentration in the range of 0.01–0.1 g/L and with the aggregate size varying from 234–343 nm. Note that for the second suspension, the transmittance of the 100× diluted samples from the original 1 g/L suspensions were measured.

In order to measure the amount of MB remaining in the solution for the discoloration tests, we measured the absorbance in the supernatant suspensions through 10 mm optical path length by UV–vis spectroscopy. The MB concentrations were calibrated from the absorbance at λ = 664 nm [19] (the calibration curve can be found in Figure S2 of Supporting Information File 1) and are plotted as a function of irradiation time.

## Results and Discussion

### Verification of the kinetic model

**Figure 3:**
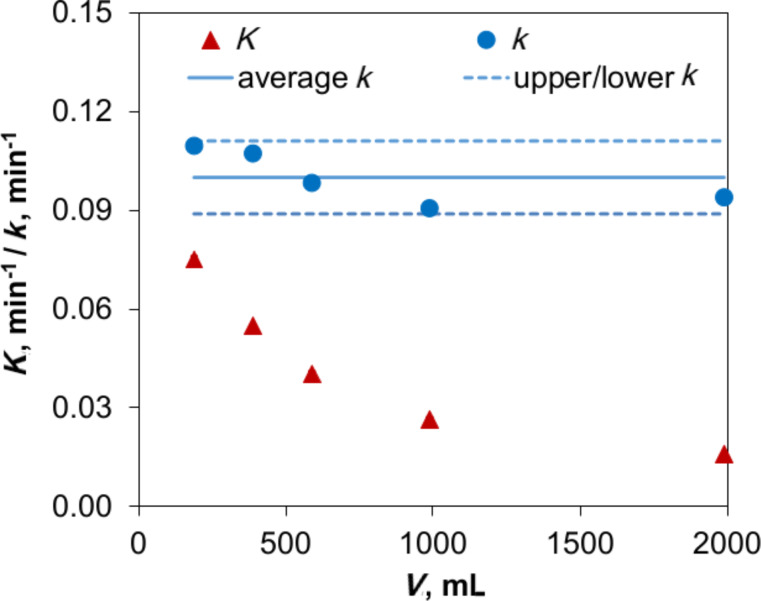
Apparent reaction rate constant *K* for the whole system and the intrinsic reaction rate constant *k* in the photoreactor, as a function of the volume of the mixing tank. Experiments were replicated twice and error bars of less than 2% were found, indicating the span between the minimum and maximum values are included in the markers and prove the precision of the data.

In essence, an increase of the suspension amount in the mixing tank, which has no contribution to the reaction, results in a slower overall degradation rate constant, *K*, for the whole system, while the reaction rate constant, *k*, in the reactor remains unchanged as expected. Experimentally the downtrend of *K* along with the variation of five suspension volumes (Figure 3) is attained.

Furthermore, the reaction rate constant, *k*, calculated from Equation 4 deviates within the upper and lower bands (Figure 3) and as predicted, yields an average value of 0.100 ± 0.011 min^−1^. This result affirms the accuracy of the model, which eventually can support further studies with the new setup based on a PFR.

### Ultrasonic dispersion of TiO_2_ aggregates

The photocatalyst P25 suspensions were dispersed with variable ultrasonic amplitudes and volumes. These different preparations allow the comparison of experiments from the aspect of electric consumption. Accordingly, the energy density, *E*_V_, defined as the integral of power consumption, *P*, by time, *t*, per volume unit, *V*, *E*_V_ = *P*·*t*/*V* was considered.

**Figure 4:**
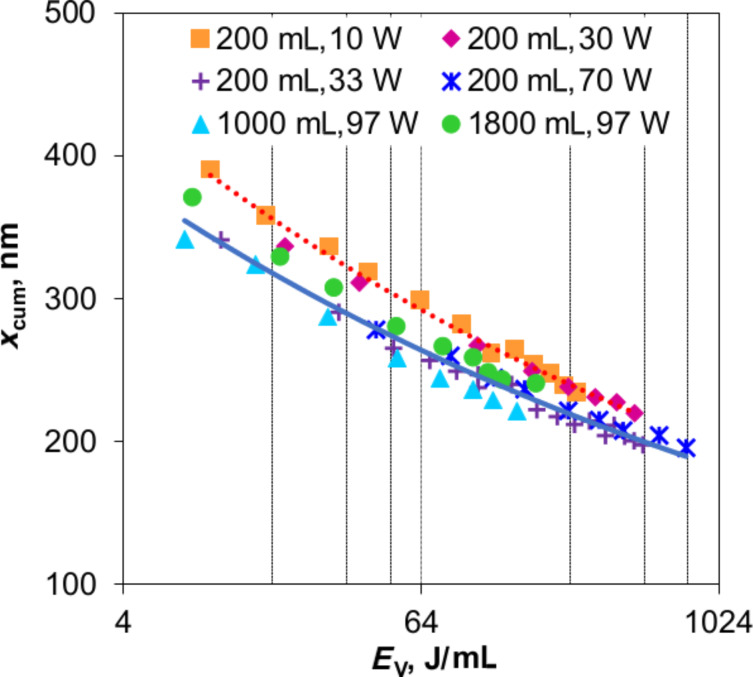
Aggregate size of P25 as a function of ultrasonic energy density performed by two ultrasonic devices, Hielscher UP100H (red dot) and Topas UDS751 (blue line).

The decrease of the intensity-weighted harmonic mean size over the energy density for independently prepared TiO_2_ P25 suspensions is shown in Figure 4. Note that the power values refer to the real electric power consumption, which is not identical to the actual input of sound energy into the suspensions. Therefore, the dispersion results slightly deviate from each other.

Experimentally, an exponential decay in photocatalyst size is specified with a high coefficient of determination (R^2^ = 0.96–0.99)

[5]
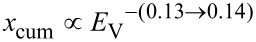


and fits to an empirical energy density concept [32]

[6]
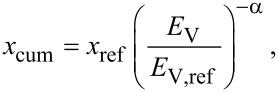


where *x*_ref_ and *E*_V,ref_ denote the corresponding reference values, and the exponent α quantifies the efficiency of the dispersion procedure (i.e., small values mean that high energy densities are required for significant changes in the size distribution).

**Figure 5:**
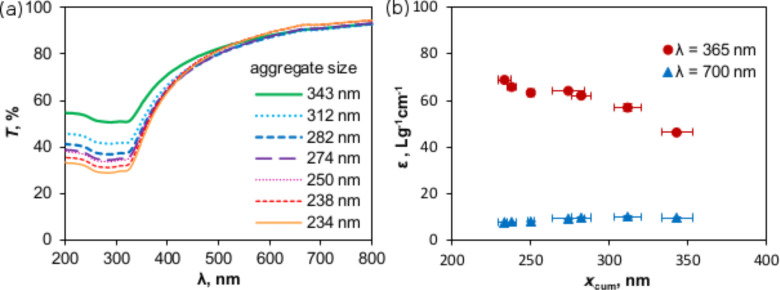
(a) Light transmittance (*T*) through a 1 cm optical path length and (b) extinction coefficient (ε) of 0.01 g/L P25 suspensions with the variation of aggregate size (measurements were repeated three times and error bars indicate the standard deviation).

Equation 5 implies that to halve the TiO_2_ P25 size requires an energy density change by a factor of 170 (corresponding to a time modification of a factor of 30 when generating a 100 W ultrasonication in a 1000 mL suspension). Consequently, this energy density concept can be used for further academic or economic estimation in the field of TiO_2_ dispersion. The influence of aggregate size on the interaction between photocatalyst and UV–vis illumination was simply tested. However, due to the very high turbidity of the original suspensions with a concentration of 1 g/L, the transmittances are extremely low for a differentiation (less than 0.16% in the UV range). The values of the 100× diluted samples are therefore substituted. As shown in Figure 5a, absorbance, scattering and other light phenomena in nano-colloidal suspensions [33] result in a loss of energy of the incident beam. In UV range, where radiation is adequate for TiO_2_ photocatalytic activation (appropriate for the photon energy of 3.2 eV and a wavelength of 387 nm [2,22,34–35]), the independence of transmittance as a function of the photocatalyst size is no longer valid as it is in the visible range. Transmittance tends to be restricted in finer suspensions as the result of a more effective absorbance [21]. A further evaluation also shows that at λ = 365 nm, disaggregation of catalyst induces a 50% higher extinction coefficient, ε, corresponding to a 1.5× increase of active sites (Figure 5b). UV irradiation can eventually penetrate the smaller aggregates in suspensions without significant obscuration.

### Degradation experiments

**Figure 6:**
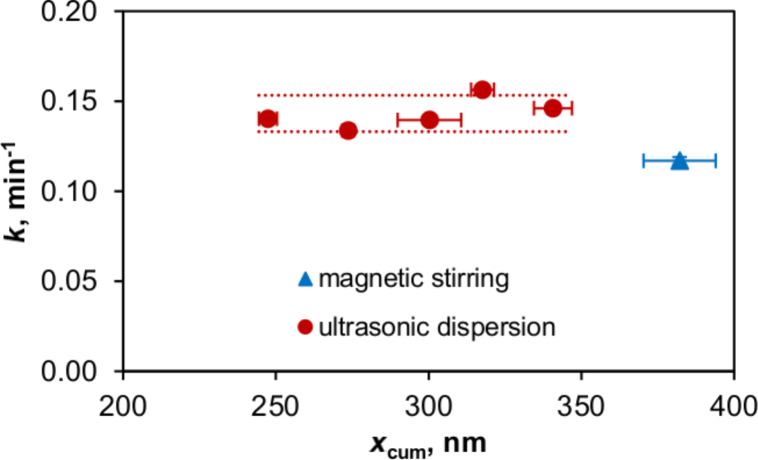
Dependence of MB discoloration in 1 g/L P25 suspensions on the photocatalyst aggregate size achieved by ultrasonic dispersion and magnetic stirring. The experiments were repeated twice, and the error bars indicate the span between the minimum and maximum values, and the upper and lower bands indicate the t-confidence intervals of 95%.

As discussed, fine aggregates in stable suspensions achieved by ultrasonic dispersion allow a more efficient photonic absorbance, and are hence expected to promote photocatalysis. Practically, a change of reaction rate constant of 1 g/L P25 suspensions by 23% is achieved when reducing *x*_cum_ from 380 to 250 nm (Figure 6), that is, the smaller particles/aggregates only have a slight influence on photocatalytic activity.

To consider the agglomerate size we approached the 90% quantile, *x*_90,int_, which is 90% of the intensity-weighted cumulative distribution. For a log–normal distribution *x*_90,int_ is derived from the median of the intensity-weighted distribution function *x*_50,int_ and the standard deviation, σ_LN_ [36–37]:

[7]
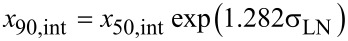


[8]
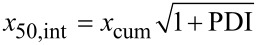


[9]
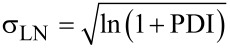


**Figure 7:**
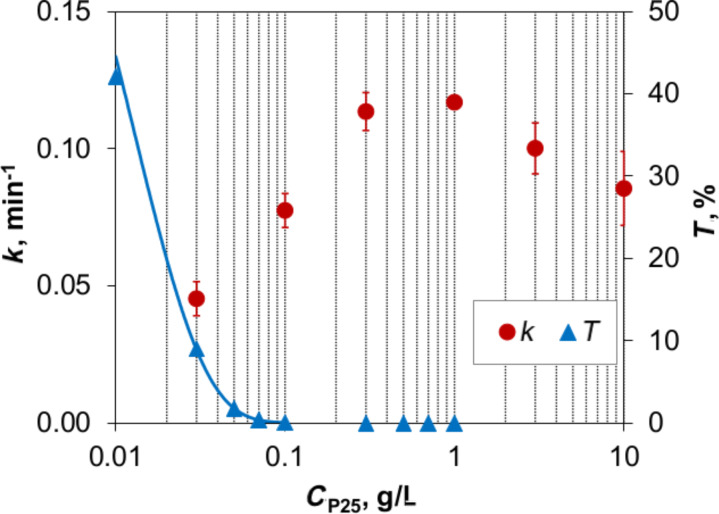
Influence of P25 catalyst concentration on the reaction rate constant and the transmittance through the thickness of the reactor at λ = 365 nm (experiments were repeated two or three times and error bars indicate the standard deviation).

The influence of agglomerate size on MB degradation given in Supporting Information File 1 shows the same tendency as that of aggregate size. Interestingly, these two results prove that photocatalytic activity is not further increased beyond a certain degree of dispersion in spite of a more efficient interaction between irradiation and photocatalyst. Note that in this research, we used 1 g/L P25 suspensions, which are considered to be the optimal concentration (Figure 7).

In the range of low concentration, the higher photocatalyst loading produces an increase of the active sites resulting in a faster degradation. A further increase in the concentration limits the photocatalytic property. This can be explained by investigating the extinction coefficient of the P25 suspension (Figure 7). At λ = 365 nm, ε = 36.65 Lg^−1^cm^−1^, which means that only 2.57% of this irradiation can penetrate a 1 mm path length of a 1 g/L suspension or a 0.1 mm path length of a 10 g/L suspension. Since the thickness of the reactor is 22 mm, very little irradiation travels through such exceedingly turbid suspensions. For this reason, the disintegration of the catalyst in the 1 g/L suspension in spite of varying the size causes the over-turbidity [5,10], resulting in the insignificant enhancement of MB discoloration.

## Conclusion

This study addressed the photocatalysis performance of suspended catalysts in an aggregated state. In particular, we examined to what degree the state of dispersion of aggregated TiO_2_ nanoparticles (P25) affects the photodegradation of methylene blue.

For this purpose, a lab-scale plug flow reactor was designed, which facilitates a defined variation of process parameters such as intensity of UV light, optical path length and flow regime. The apparent reaction rate constant of such a reactor can be easily translated into an intrinsic reaction rate constant when the material balance between the storage tank and the reactor is established. The proposed calculation scheme was experimentally verified.

In addition, the study took a closer look at the dispersion procedures for the TiO_2_ suspensions. Focus was placed on ultrasonication, which yields highly intense hydrodynamic and thermal stresses and thus allows for a significant disintegration of particle aggregates. We showed that the energy density concept works well for comparing the dispersion performance of different ultrasonic processors and facilitates the comparison of dispersion procedures between different laboratories.

The central part of the paper was the study of the discoloration of MB in the presence of P25 under UV illumination. The size of the aggregates appeared to have only a minor influence on the intrinsic reaction rate constant, even though the efficiency of photon absorbance increases with further dispersion. Obviously, the maximum reaction rate is already achieved after short ultrasonication time, which disperses the large micrometer-sized agglomerates into submicron aggregates. This outcome is explained by the limited UV penetration depth into the concentrated catalyst suspensions. In this regard it may not be surprising, yet from a practical point of view, it may help to reduce the energy consumption in the preparation of photocatalyst suspensions and to optimize their total particle concentration.

## Supporting Information

Details of a model for determination of the reaction rate constant in the experimental setup based on a PFR, the calibration of MB, the stability test of suspensions, the results of photon correlation spectroscopy, and the impact of agglomerate size on the discoloration of MB in photocatalysis can be found in this file.

File 1 Details for the reaction rate model and experimental setup
