# Supplementary material for: Impact of ultrasonic dispersion on the photocatalytic activity of titania aggregates
Source: Beilstein J Nanotechnol. 2015 Dec 17;6:2423–30. doi: 10.3762/bjnano.6.250 (PMC4685913; doi:10.3762/bjnano.6.250)
Supplement: File 1 — Details for the reaction rate model and experimental setup [file Beilstein_J_Nanotechnol-06-2423-s001.pdf]

Supporting information

for

Impact of ultrasonic dispersion on the photocatalytic activity of titania aggregates

Hoai Nga Le*[1,2], Frank Babick[3], Klaus Kühn[1], Minh Tan Nguyen[2], Michael Stintz[3] and Gianaurelio Cuniberti[1,4,5]

[1]Institute for Materials Science and Max Bergmann Center of Biomaterials, TU Dresden, 01062 Dresden, Germany, [2]School of Chemical Engineering, Hanoi University of Science and Technology, 10000 Hanoi, Vietnam, [3]Institute of Process Engineering and Environmental Technology, TU Dresden, 01069 Dresden, Germany, [4]Dresden Center for Computational Materials Science (DCCMS), TU Dresden, 01062 Dresden, Germany and [5]Center for Advancing Electronics Dresden, TU Dresden, 01062 Dresden, Germany

Email: Hoai Nga Le - hnle@nano.tu-dresden.de

* Corresponding author

Details for the reaction rate model and experimental setup

Determination of reaction rate constant in the experimental setup based on a plug flow reactor

Nomenclature

C_{ini}, C_0	concentration of species A prior to the reaction and at time t, [mol/m^3].
k	reaction rate constant of the reactor, [s^{-1}].
K	overall degradation constant, [s^{-1}].
n	moles of species A, [mol].
$\dot{n}$	molar flow rate of species A, [mol/s].
r	reaction rate, [mol/m^{3}s].
t	time, [s].
V	volume, [m^3].
R,T,M	the subscripts designate for the plug flow reactor, tubing, and the mixing tank.
$\dot{v}$	flow rate, [m^3/s].
ϑ	stoichiometric coefficient of species A, dimensionless.

Assumptions

The model development is based on the following assumptions:

- There is no axial batch mixing in the plug flow reactor (PFR). Variations only exist along the length of the reactor.

- In the PFR, the process is assumed as a steady-state isothermal reaction.
- Since the reaction is slow and dominates over the mass transfer, the kinetic of PFR is considered by means of a pseudo-homogenous model.
- Reaction occurs only in the PFR where photocatalyst is activated by absorbing photon energy of UV-irradiation and it is a first-order reaction

$$A \xrightarrow[h\nu]{cat.} B$$

- There is no reaction in the mixing tank.
- The mixing in the tank is assumedly ideal.

Modeling

Consider a volume element of species A in the PFR, the material balance follows

$$\frac{d\dot{n}_1}{dV_R} = \vartheta r \quad \text{(S1)}$$

Since the reaction is first-order, $\vartheta = -1$, and $r = kC_1$. The integral gives the solution as

$$C_0(t) = C_0(t - t_R)e^{-kt_R} \quad \text{(S2)}$$

Material balance for species A in the mixing tank is described as

$$\frac{dn_0}{dt} = -\dot{n}_0 + \dot{n}_2 \quad \text{(S3)}$$

By substituting with the concentrations of species A and letting $\tau = t_T + t_R$, the material balance in the whole setup becomes a linear delay differential equation

$$\frac{dC_0}{dt}t_M = -C_0(t) + C_0(t - \tau)e^{-kt_R} \quad \text{(S4)}$$

Try the general exponential solution $C_0(t) = Ae^{-Kt}$, Eq.(S4) is derived as

$$1 - Kt_M = e^{K\tau - kt_R} \quad \text{(S5)}$$

Eq.(S5) is the characteristic equation and the number of characteristic solutions is determined by looking for the intersection(s) of plotting curves. For positive t_M and τ, there is one intersection (Figure S1), correspondent with one single solution K equaling to $C_0 = Ae^{-Kt}$.

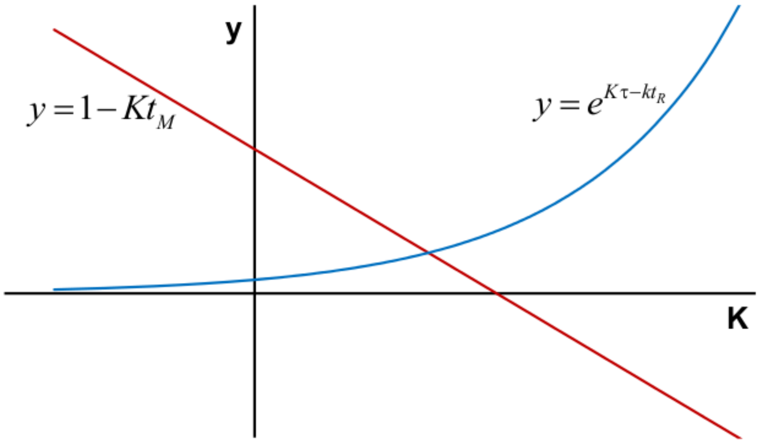

Figure S1: Solution of the characteristic equation.

The boundary condition is determined at $t = 0, C_0(t) = C_{ini}$. Accordingly, the conversion of species A follows

$$C_0(t) = C_{ini} e^{-Kt} \tag{S6}$$

and the reaction rate constant k is investigated as

$$k = \frac{K(t_T + t_R) - \ln(1 - Kt_M)}{t_R} \tag{S7}$$

Calibration curve of methylene blue measured by UV–vis spectroscopy

Methylene blue (MB) (Merck KGaA) 3.126 mM was used as the stock solution for the calibration. The eight concentrations ranging from 0.537−12.344 μM were prepared. The absorbance of solutions was scanned in the wavelength of 200−800 nm through 10 mm optical path length by a Varian Cary 100 Bio spectrometer. The maximum absorbance at wavelength λ = 664 nm was picked out from the whole UV-Vis spectra. All measurements were repeated three times. Analysis of the values along with MB concentrations were shown in Figure S2, where standard deviations of each point are too small to be observed, lower and upper CI respectively show the 95% confidence intervals of the linear fit.

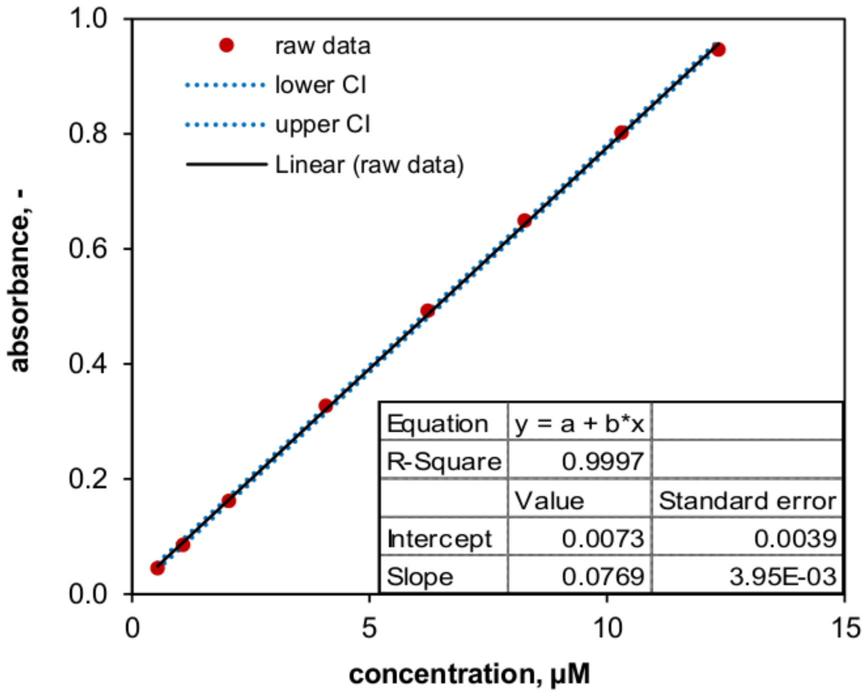

Figure S2: Calibration of methylene blue.

Ultrasonic dispersion of P25 aggregates

The 1 g/L P25 suspensions were disintegrated by ultrasonication. Aggregate sizes were examined as a function of energy density E_V (Table S1–Table S6). The size properties were measured by the photon correlation spectroscopy (PCS) Malvern HPPS-ET, including intensity-weighted harmonic mean size x_{cum}, polydispersity index PDI, the median of the intensity-, number- and volume-weighted distribution function $x_{50,\text{int}}$, $x_{50,0}$, and $x_{50,3}$. The cumulative sizes $x_{cum}{}^{(*)}$ comparable to measurement by PCS Malvern Nano S90 were converted from x_{cum} as

$$x_{cum}{}^{(*)} = 1.01 \times x_{cum} \times t^{0.027} \quad \text{(S7)}$$

where parameters were determined by correlating measurements along with time t by two instruments (HPPS-ET and Nano S90).

Table S1: Ultrasonic dispersion of 200 mL P25 suspensions by Hielscher UP100H with a generating power of 10 W.

No	E_V, J/mL	x_{cum}, nm	PDI, -	$x_{50,int}$, nm	$x_{50,0}$, nm	$x_{50,3}$, nm	$x_{cum}{}^{(*)}$, nm
1	9	383	0.499	446	131	530	392
2	15	351	0.437	397	131	702	366
3	27	326	0.389	369	113	435	347
4	39	305	0.370	326	112	352	328
5	63	279	0.337	300	99	219	304
6	93	263	0.300	277	93	181	290
7	123	242	0.295	262	121	179	269
8	153	244	0.241	268	98	149	273

9	183	233	0.240	254	102	143	262
10	213	226	0.224	245	95	138	255
11	243	218	0.225	234	105	136	247
12	273	213	0.222	224	109	143	242

Table S2: Ultrasonic dispersion of 200 mL P25 suspensions by Hielscher UP100H with a generating power of 30 W.

No	E_V, J/mL	x_{cum}, nm	PDI, -	$x_{50,int}$, nm	$x_{50,0}$, nm	$x_{50,3}$, nm	$x_{cum}^{(*)}$, nm
1	18	329	0.419	357	114	482	337
2	36	298	0.397	311	105	367	311
3	108	249	0.270	262	108	187	268
4	180	229	0.249	248	93	141	250
5	252	216	0.240	225	110	145	238
6	324	209	0.224	222	102	135	231
7	396	204	0.222	216	99	135	227
8	468	197	0.181	214	91	122	220

Table S3: Ultrasonic dispersion of 200 mL P25 suspensions by Topas UDS751 with a generating power of 33 W.

No	E_V, J/mL	x_{cum}, nm	PDI, -	$x_{50,int}$, nm	$x_{50,0}$, nm	$x_{50,3}$, nm	$x_{cum}^{(*)}$, nm
1	10	333	0.396	370	111	597	335
2	30	276	0.373	304	118	255	286
3	50	252	0.296	260	130	205	265
4	69	242	0.277	249	127	218	256

5	89	233	0.265	235	127	211	249
6	109	221	0.235	232	122	166	237
7	109	229	0.241	247	105	143	246
8	149	221	0.238	232	113	151	239
9	188	204	0.228	214	123	151	222
10	228	198	0.229	209	118	145	217
11	267	192	0.224	207	115	140	211
12	307	194	0.196	207	98	126	214
13	356	184	0.203	198	109	131	204
14	386	188	0.209	195	123	142	209
15	426	183	0.196	188	121	138	204
16	465	179	0.208	190	116	136	200
17	505	176	0.192	184	121	137	197

Table S4: Ultrasonic dispersion of 200 mL P25 suspensions by Topas UDS751 with a generating power of 70 W.

No	E_V, J/mL	x_{cum}, nm	PDI, -	$x_{50,int}$, nm	$x_{50,0}$, nm	$x_{50,3}$, nm	$x_{cum}^{(*)}$, nm
1	42	267	0.277	292	105	216	274
2	84	244	0.271	260	114	171	255
3	126	226	0.245	245	103	144	239
4	168	217	0.216	238	88	133	231
5	252	202	0.197	219	99	131	217
6	336	194	0.190	205	108	133	210
7	420	186	0.182	200	98	125	203
8	588	183	0.168	194	103	128	201
9	756	175	0.149	187	103	124	194

Table S5: Ultrasonic dispersion of 1000 mL P25 suspensions by Topas UDS751 with a generating power of 97 W.

No	E_V, J/mL	x_{cum}, nm	PDI, -	$x_{50,int}$, nm	$x_{50,0}$, nm	$x_{50,3}$, nm	$x_{cum}^{(*)}$, nm
1	7	342	0.485	345	107	319	350
2	14	316	0.388	337	106	338	330
3	27	277	0.354	288	106	251	295
4	51	245	0.268	260	96	152	265
5	76	229	0.255	246	98	142	251
6	104	220	0.232	237	96	134	243
7	125	212	0.209	232	96	131	235
8	156	204	0.199	225	89	124	228

Table S6: Ultrasonic dispersion of 1800 mL P25 suspensions by Topas UDS751 with a generating power of 97 W.

No	E_V, J/mL	x_{cum}, nm	PDI, -	$x_{50,int}$, nm	$x_{50,0}$, nm	$x_{50,3}$, nm	$x_{cum}^{(*)}$, nm
1	8	370	0.446	379	111	323	379
2	17	322	0.418	323	114	417	336
3	28	297	0.398	290	98	206	316
4	51	266	0.328	293	93	152	288
5	78	250	0.291	272	100	151	274
6	104	241	0.256	264	78	144	266
7	119	230	0.237	247	105	149	255
8	136	225	0.231	248	89	131	251
9	186	220	0.219	241	104	138	247

Stability of dispersed suspensions

The stability of the P25 titania suspensions (at room temperature) were examined by monitoring the size parameters over a time period of three days. Their aggregate sizes measured by PCS Malvern Nano S90 including x_{cum}, PDI, $x_{50,\text{int}}$, $x_{50,0}$, and $x_{50,3}$ (Table S7) show that a stable suspension can be achieved by ultrasonication, while the re-aggregation of the colloids is induced by a conventional stirring.

Table S7: Aggregate size of P25 photocatalyst in MB solution with and without ultrasonic dispersion after three day in room conditions.

No	dispersion	time, day	x_{cum}, nm	PDI, -	$x_{50,int}$, nm	$x_{50,0}$, nm	$x_{50,3}$, nm
1	magnetic stirring	0	390	0.396	476	198	892
2	magnetic stirring	1	447	0.480	395	229	834
3	magnetic stirring	2	418	0.432	484	199	790
4	magnetic stirring	3	432	0.390	518	228	866
5	ultrasonic dispersion	0	219	0.166	240	129	238
6	ultrasonic dispersion	1	220	0.175	237	144	258
7	ultrasonic dispersion	2	233	0.177	257	140	365
8	ultrasonic dispersion	3	223	0.190	243	133	252

Color removal of MB in P25 suspensions

The photocatalytic properties of P25 were examined by the discoloration of MB. Photocatalyst aggregate sizes were measured by PCS Malvern Nano S90 including x_{cum}, PDI, $x_{50,\text{int}}$, $x_{50,0}$, and $x_{50,3}$. The 90% quantile of intensity-weighted cumulative distribution $x_{90,\text{int}}$ calculated as Eq. (7) is considered as agglomerate size of photocatalyst. The apparent reaction rate constant K in the whole setup and the intrinsic reaction rate constant k in the reactor are calculated as Eq. (3) and Eq.(4). Data are given in Table S8 and Figure S3.

Table S8: Data of MB discoloration in 1 g/L P25 suspensions.

No	x_{cum}, nm	PDI, -	$x_{50,int}$, nm	$x_{50,0}$, nm	$x_{50,3}$, nm	$x_{90,int}$, nm	K, min^{-1}	k, min^{-1}
1	245	0.218	265	102	343	478	0.096	0.140
2	250	0.229	264	120	446	494	0.096	0.140
3	272	0.258	278	169	340	564	0.091	0.133
4	275	0.249	287	112	386	562	0.092	0.134
5	293	0.285	307	148	431	631	0.095	0.139
6	308	0.352	319	149	360	708	0.096	0.140
7	315	0.340	338	109	404	743	0.106	0.155
8	320	0.353	335	123	400	754	0.108	0.158
9	336	0.341	369	125	499	780	0.100	0.146
10	345	0.386	334	156	581	845	0.100	0.146
11	374	0.368	386	160	601	845	0.081	0.118
12	391	0.431	401	185	729	1006	0.079	0.115

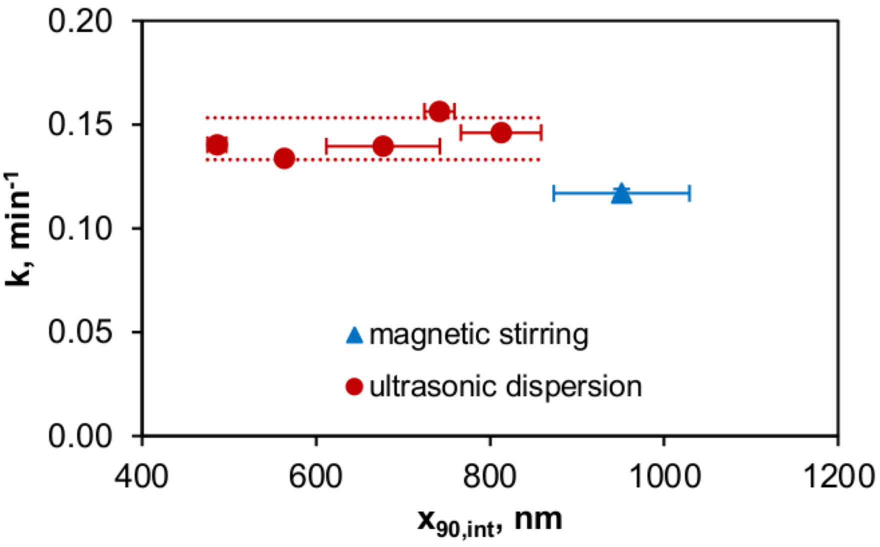

Figure S3: Dependency of MB discoloration in 1 g/L P25 suspensions on the photocatalyst agglomerate size achieved by ultrasonic dispersion and magnetic stirring (experiments were repeated twice, error bars indicate the span between the minimum and maximum values, and the upper and lower bands indicate the t-confidence intervals of 95%).
